# Thrombotic Microangiopathy with Skin Localization Secondary to Cytarabine-Daunorubicin Association: Report of a Case

**DOI:** 10.1155/2012/806476

**Published:** 2012-05-07

**Authors:** S. Regragui, S. Amelal, S. Astati, M. Zine, N. Alami Drideb, A. Al Bouzidi, N. Messaoudi, M. Benkirane, K. Doghmi, M. Mikdame

**Affiliations:** ^1^Department of Clinical Hematology, Military Hospital Mohammed V, Rabat, Morocco; ^2^Department of Pathology, Military Hospital Mohammed V, Rabat, Morocco; ^3^Department of Biological Hematology, Military Hospital Mohammed V, Rabat, Morocco; ^4^Blood Transfusion Centre, Military Hospital Mohammed V, Rabat, Morocco

## Abstract

The thrombotic microangiopathy is a syndrome characterized by the combination of mechanical hemolytic anemia, peripheral thrombocytopenia, and organ failure of variable severity. In addition to the idiopathic form, several cases are identified as secondary to pregnancy, infections, disease systems, organ transplants, and cancer. Other forms are secondary to drugs including antimitotics. We report the case of a patient followed for acute myelogenous leukemia. She received induction chemotherapy combining daunorubicin and cytarabine, complicated by thrombotic thrombocytopenic purpura.

## 1. Introduction

 The term thrombotic microangiopathy (TMA) includes different pathologies which are characterized by the association of mechanical hemolytic anemia and thrombocytopenia. The TMA syndrome is defined histologically by the presence of thrombi in terminal arterioles and capillaries, responsible for visceral pain of variable severity. This syndrome can develop life-threatening, requiring to know the early recognition and to organize a specialized care in emergency. The thrombotic microangiopathies include thrombotic thrombocytopenic purpura (TTP), hemolytic uremic syndrome (HUS), and related syndromes known as disseminated intravascular coagulation (DIC), the heparin-induced thrombocytopenia (HIT), some forms of the syndrome antiphospholipid, and hemolysis, elevated liver enzymes and low platelet (HELLP) syndrome. Those syndromes may be idiopathic or secondary to hematopoietic stem cell transplantation, infection with human immunodeficiency virus (HIV), malignant hypertension, certain cancers, and chemotherapies.

## 2. Observation

**Figure 1:**
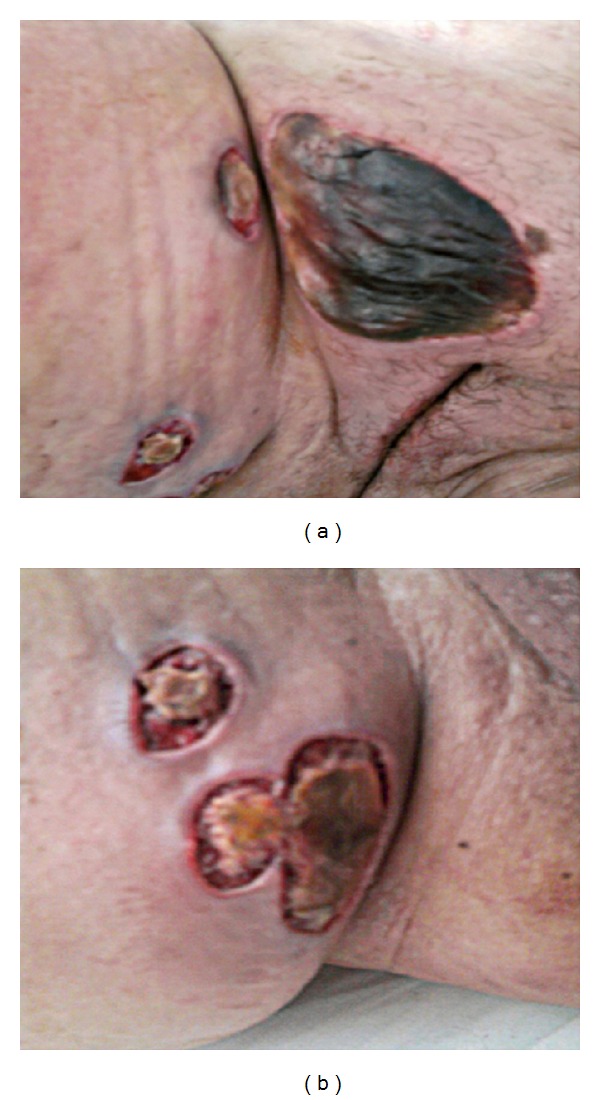
Skin necrosis in the d12 of induction daunorubicin-cytarabine.

This is a 31-year-old patient without medical history. In November 2010, she presented with acute myelogenous leukemia 2 (LAM2) not tumoral and pancytopenic with *t*(8,21) and del X. She received induction chemotherapy combining daunorubicin (80 mg/m² de D1 to D3) and cytarabine (200 mg/m² de D1 to D7). At D8 of treatment and during postchemotherapy aplasia, the patient has impaired consciousness with a Glasgow Coma Score at 12; following the transfusion of apheresis platelet pellets (APP), the symptoms have spontaneously regressed after 24 h, and brain scan came back normal. 4 days later, the patient developed a skin necrosis in the pelvis and internal faces of thighs (Figures 1(a) and 1(b)) with mucocutaneous haemorrhagic syndrome made of bloody sputum, spontaneous ecchymoses on both legs, and petechial purpura of the neck and trunk, all operating in a context of dyspnea, fever among 39°C, and hemodynamic stability. The chest scan objectives bilateral interstitial syndrome with suspected diffuse lung disease. The treatment with ceftazidime, vancomycin, voriconazole, and aciclovir was instituted. Blood cultures were sterile. However, she remained febrile.

**Figure 2:**
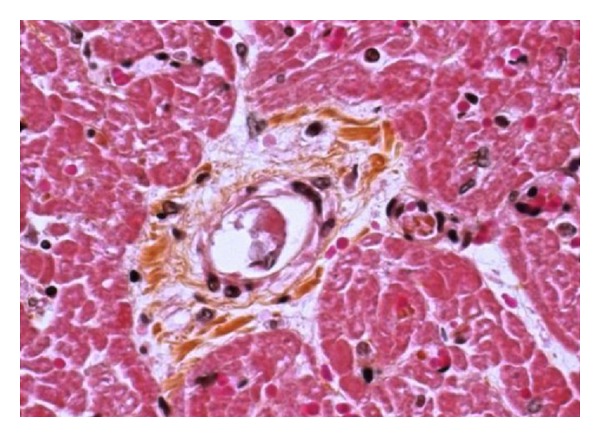
(High magnification) fibrinoid necrosis of vessel walls with perivascular myxoid degeneration.

 The complete blood count (CBC) objectives aplasia with profound agranulocytosis, normochromic normocytic anemia at 7.2 g/dL, and thrombocytopenia at 17 Giga/L. She manifested accelerated consumption of transfused platelets while remaining severely thrombocytopenic. The blood smear has objectified schizocytosis at 10%, and the crasis was normal many times, thus eliminating consumptive coagulopathy. The initial laboratory testing carried out in front of this mechanical anemia with thrombocytopenia unresponsive to transfusions showed an increased LDH 863 UI/l (2 times normal), serum potassium to 4.2 mmol/l (3–5 mmol/l), collapsed serum haptoglobin (0.53 g/l), hyperbilirubinemia (25 mg/l), the direct Coombs test (the test in antiglobinul) negative, proteinuria 900 mg/24 h, and renal function normal with serum creatinine at 5 mg/l (7–14 mg/l). The histopathological study of these lesions showed multiple intravascular thrombi. The affected vessels were small arterioles occluded by pale eosinophilic material. Some of the vessels showed swelling of the endothelium. The focal areas of the thrombi were composed of fibrin, whereas the major portion of these thrombi were metachromatic, indicating that they were mainly composed of platelets which characterizes TMA (Figure 2).

 The therapeutic management consisted in a combination of corticosteroids, daily plasma exchanges, and four weekly infusions of Rituximab in first line. The outcome was favorable with regression of skin lesions, correction of thrombocytopenia, the disappearance of schizocytes, and normalization of LDH. Acetyl salicylic acid was introduced when the platelet count reached 50.000/mm^3^, and the plasma exchanges were stopped when the platelet count reached 150.000/mm^3^. The patient was discharged from aplasia on day 28, and she is declared in full remission. Two cures of consolidation by high-dose cytarabine are administered, but this time without incident.

## 3. Discussion

 Thrombotic thrombocytopenic purpura (TTP) is a thrombotic microangiopathy (TMA) historically associated with five major events reported in 1924 by Moschowitz which are mechanical hemolytic anemia, thrombocytopenia, fever, and renal and neurological disorders [1, 2]. Currently, in clinical practice, the triad of thrombocytopenia, a blood smear to schizocytosis (1–4% or more of total erythrocytes) and an increase in LDH level, is sufficient to suggest the diagnosis [3, 4]. Coagulation tests are usually normal. Note that fever is present in 59–98% of cases. It could be related to the release of pyrogenic substances in tissue ischemia, or an infectious process. Neurological impairment was observed in 84–92% of cases. It is characterized by its sudden onset and transience. Renal failure is found in nearly half of the cases; in other cases, the kidney can be summarized as proteinuria whose flow is usually less than 3 g/24 h, or hematuria [5]; this is the case of our patient. Many studies suggest that an attack on the vascular endothelium is an early phenomenon in the onset of an episode of TMA. This attack results in activation and/or injury of endothelial cells. Precipitating factors behind this attack can be many: various infections, drugs, cancer, or transplant. This endothelial activation/injury contributes to the development of platelet hyperaggregability, favoring the formation of platelet thrombi in the microcirculation [5], these microthrombi are responsible for hypoperfusion of the organs affected and hemolytic anemia known as “mechanical” [3, 6]. The predominant consumption of platelets, with evidence of minimal fibrinogen depletion, and the platelet-derived nature of the microvascular lesions, characterize TTP [7].

 The association between TMA and malignant diseases covers two distinct clinical conditions, the first is the occurrence of microangiopathic hemolytic anemia complicating metastatic adenocarcinomas, most of which are mucus-secreting tumors [8]. In the second, more common, the TMA is the waning of chemotherapy, while tumor disease is in remission, the case in which the kidney is often predominant, producing HUS. Mitomycin C is the drug most frequently incriminated [9–11]. The incidence of HUS complicating treatment with mitomycin varies from 2 to 10% and is related to the cumulative dose. The clinical and histological features of TMA associated with mitomycin (and gemcitabine) and its mechanism of injury are well documented [12–14].

 Other antimitotic agents have been implicated, including cisplatin [15], deoxycoformycin [16], regimens containing cisplatin, bleomycin and vinca alkaloids [17], combination therapy of cisplatin [17], and the combination of daunorubicin and cytosine arabinoside [7]; as in our patient, however, only one similar case was reported in the literature in 1986. She was a woman in complete remission from acute myeloblastic leukemia that developed thrombotic thrombocytopenic purpura (TTP) subsequent to the third intensive consolidation cycle of cytosine arabinoside and daunorubicin chemotherapy. The constellation of clinical manifestations indicative of TTP was recognized only in retrospect, as they were initially attributed to more usual complications of bone-marrow-ablative chemotherapy. The manifestations, probably fueled by numerous red cell and platelet transfusions, increased at the time of recovery of hematopoiesis. At postmortem examination, characteristic microvascular lesions were found in most organs [7]. The mechanism of TMA in post chemotherapy other than mitomycin C is unclear. It is not yet clear whether an excessive release of a similar or more antiangiogenic soluble receptors of neoplastic cells can cause a thrombotic microangiopathy in some patients after chemotherapy in malignancies [4]. Recall that the deficiency of ADAMTS13 (*a disintegrin and metalloproteinase with thrombospondin type 1 motif a, member 13*), a metalloprotease that specifically cleaves the mega-multimers of von Willebrand factor in multimers with low molecular weight, is involved in most of idiopathic TTP [5]. This deficit is severe and less than 5% of the normal activity of ADAMTS13, whereas it is moderate or nonexistent in the secondary forms, including chemotherapy [18].

 The treatment of TTP is always an emergency. In adults, treatment is currently based on the achievement of plasma exchange (PEX) [19, 20]. If they cannot be made in emergency, infusions of large volumes of plasma (30 mL/kg/j) may be started [21, 22]. Treatment is continued until the stabilization of normal count of platelet (more than 150 × 109/l) for at least 48 hours [5]. We must ensure that the reticulocyte count and LDH are currently declining. The duration of treatment can be very variable. The decrease in the pace of PEX should be gradual and should be monitored by signs of recurrence, which should motivate further the achievement of daily plasma exchange. Corticosteroids in high doses have been reported as effective in 56% of TTP purely hematological [19]. However, there is no randomized study to clearly demonstrate their effectiveness. Although the level of evidence is so weak, a steroid methylprednisolone (1 mg/kg/j for 3 weeks with tapering) should be discussed, in the absence of indication cons. Their administration is the waning of immediate plasma exchange. Antiplatelet agents are widely used because it exists in the TMA a platelet hyperaggregability [23]. But they increase the risk of bleeding [24]. They are usually introduced when the platelet count is greater than 50 × 109/l [5]. Other treatments such as infusions of heparin, fibrinolytic, prostacyclin, or vitamin E are unnecessary and sometimes dangerous [25].

 The relapse can occur in 30% of cases in the acute phase. In these patients, interesting results have been reported with the use of monoclonal antibodies directed against the CD20 antigen on B lymphocytes (Rituximab) [26].

 A recent British study in Phase II showed that the weekly use of rituximab in combination with standard therapy (EP and steroids) on the front line was safe, effective, and well tolerated. This is the case in our patient who benefited from this association in the front line with achieving a complete remission. This study showed a significant reduction in hospital stay. Further, globally, there is a reduction (not significant) of the average number of treatment by PEX until remission; this reduction was significant among white patients. The relapse rate was also significantly reduced compared with controls and with published data in the literature. The relapse rate was associated with a reduction in the rate of IgG anti-ADAMTS13 and an increase in ADAMTS13 activity. The reduction of relapse rate suggests that the anti-CD20 therapy halt the production of anti-ADAMTS13, enabling sustained remission. Maximizing therapy for patients with clinical signs of organ dysfunction in advanced stages, particularly cardiac and neurological, with intensive treatment by the PEX, steroids, and early use of rituximab accelerates complete remission and prevents recurrent relapses [27].

## 4. Conclusion

 The thrombotic microangiopathy is a rare complication of chemotherapy in which the diagnosis is very difficult due to the nonspecific symptoms, but we must think about in order to establish a specific treatment time. The use in the first line of Rituximab in combination with standard therapy could improve the management of this disease whose prognosis is poor.

## References

[1] Moschcowitz E (1925). An acute febrile pleiochromic anemia with hyaline thrombosis of the terminal arterioles and capillaries. *Archives of Internal Medicine*.

[2] Amorosi EL, Ultmann JE (1966). Thrombotic thrombocytopenic purpura: report of 16 cases and review of the literature. *Medicine*.

[3] Moake JL (2002). Thrombotic microangiopathies. *New England Journal of Medicine*.

[4] Moake J (2009). Thrombotic thrombocytopenia purpura (TTP) and other thrombotic microangiopathies. *Best Practice and Research*.

[5] Coppo P, Vernant JP, Veyradier A (2005). Thrombotic thrombocytopenic purpura and other thrombotic microangiopathy syndromes. *EMC—Hematologie*.

[6] Ruutu T, Barosi G, Benjamin RJ (2007). Diagnostic criteria for hematopoietic stem cell transplant-associated microangiopathy: Results of a consensus process by an International Working Group. *Haematologica*.

[7] Byrnes JJ, Baquerizo H, Gonzalez M, Hensely GT (1986). Thrombotic thrombocytopenic purpura subsequent to acute myelogenous leukemia chemotherapy. *American Journal of Hematology*.

[8] Murgo AJ, Kaplan BS, Trompeter RS, Moake JL (1992). Cancer and chemotherapy associated thrombotic microangiopathy. *Hemolytic Uremic Syndrome and Thrombotic Thrombocytopenic Purpura*.

[9] Giroux L, Bettez P, Giroux L (1985). Mitomycin-C nephrotoxicity: a clinico-pathologic study of 17 cases. *American Journal of Kidney Diseases*.

[10] Liu K, Mittelman A, Sproul EE, Elias EG (1971). Renal toxicity in man treated with mitomycin C. *Cancer*.

[11] Nagaya S, Wada H, Oka K (1995). Hemostatic abnormalities and increased vascular endothelial cell markers in patients with red cell fragmentation syndrome induced by mitomycin C. *American Journal of Hematology*.

[12] Cordonnier D, Vert-Pre FC, Bayle F (1985). Nephrotoxicity of mitomycin C (apropos of 25 case reports). Results of a multicenter survey organized by the Society of Nephrology. *Nephrologie*.

[13] Cattell V (1985). Mitomycin-induced hemolytic uremic kidney. An experimental model in the rat. *American Journal of Pathology*.

[14] Duperray A, Tranqui L, Alix J (1984). The effect of mitomycin C on the biosynthesis of prostacyctin by primary cultures of human umbilical lord vein endothelial cells. *Proceedings of the International Congress of Nephrology*.

[15] Canpolat C, Pearson P, Jaffe N (1994). Cisplatin-associated hemolytic uremic syndrome. *Cancer*.

[16] Leach JW, Pham T, Diamandidis D, George JN (1999). Thrombotic thrombocytopenic purpura—Hemolytic uremic syndrome (TTP-HUS) following treatment with deoxycoformycin in a patient with cutaneous T- cell lymphoma (sezary syndrome): a case report. *American Journal of Hematology*.

[17] Jackson AM, Rose BD, Graff LG (1984). Thrombotic microangiopathy and renal failure associated with antineoplastic chemotherapy. *Annals of Internal Medicine*.

[18] Levy GG, Nichols WC, Lian EC (2001). Mutations in a member of the ADAMTS gene family cause thrombotic thrombocytopenic purpura. *Nature*.

[19] Bell WR, Braine HG, Ness PM, Kickler TS (1991). Improved survival in thrombotic thrombocytopenic purpura-hemolytic uremic syndrome—clinical experience in 108 patients. *New England Journal of Medicine*.

[20] Rock GA, Shumak KH, Buskard NA (1991). Comparison of plasma exchange with plasma infusion in the treatment of thrombotic thrombocytopenic purpura. *New England Journal of Medicine*.

[21] Coppo P, Bussel A, Charrier S (2003). High-dose plasma infusion versus plasma exchange as early treatment of thrombotic thrombocytopenic purpura/hemolytic-uremic syndrome. *Medicine*.

[22] Novitzky N, Jacobs P, Rosenstrauch W (1994). The treatment of thrombotic thrombocytopenic purpura: Plasma infusion or exchange?. *British Journal of Haematology*.

[23] del Zoppo GJ (1987). Antiplatelet therapy in thrombotic thrombocytopenic purpura. *Seminars in Hematology*.

[24] Rosove MH, Ho WG, Goldfinger D (1982). Ineffectiveness of aspirin and dipyridamole in the treatment of thrombotic thrombocytopenic purpura. *Annals of Internal Medicine*.

[25] Gaddis TG, Guthrie TH, Drew MJ, Sahud M, Howe RB, Mittelman A (1997). Treatment of plasma refractory thrombotic thrombocytopenic purpura with protein a immunoabsorption. *American Journal of Hematology*.

[26] Yomtovian R, Niklinski W, Silver B, Sarode R, Tsai HM (2004). Rituximab for chronic recurring thrombotic thrombocytopenic purpura: A case report and review of the literature. *British Journal of Haematology*.

[27] Scully M, McDonald V, Cavenagh J (2011). A phase 2 study of the safety and efficacy of rituximab with plasma exchange in acute acquired thrombotic thrombocytopenic purpura. *Blood*.

